# Gene expression analysis of a murine model with pulmonary vascular remodeling compared to end-stage IPAH lungs

**DOI:** 10.1186/1465-9921-13-103

**Published:** 2012-11-17

**Authors:** Kayoko Shimodaira, Yoichiro Okubo, Eri Ochiai, Haruo Nakayama, Harutaka Katano, Megumi Wakayama, Minoru Shinozaki, Takao Ishiwatari, Daisuke Sasai, Naobumi Tochigi, Tetsuo Nemoto, Tsutomu Saji, Katsuhiko Kamei, Kazutoshi Shibuya

**Affiliations:** 1 Department of Surgical Pathology, Toho University School of Medicine, 6-11-1 Omori-Nishi, Ota-Ku, Tokyo, 143-8541, Japan; 2 Department of Pediatrics, Toho University Omori Medical Center, 6-11-1 Omori-Nishi, Ota-Ku, Tokyo, 143-8541, Japan; 3 Department of Pathogenic Fungi, Medical Mycology Research Center, Chiba University, 1-8-1 Inohana, Chuo-ku, Chiba, 260-8673, Japan; 4 Department of Microbiology, Immunology and Molecular Genetics, University of Kentucky College of Medicine, 800 Rose Street, Lexington, KY, 40536, USA; 5 Department of Pathology, National Institute of Infectious Diseases, 1-23-1 Toyama, Shinjuku-ku, Tokyo, 162-8640, Japan; 6 Department of Dermatology, Peking University First Hospital, NO.7, Xishiku Street, Xicheng District, Beijing, 100034, China

**Keywords:** Pulmonary Vascular Remodeling, *Stachybotrys chartarum*, BMP signaling, BMPR2, PCP pathway

## Abstract

**Background:**

Idiopathic pulmonary arterial hypertension (IPAH) continues to be one of the most serious intractable diseases that might start with activation of several triggers representing the genetic susceptibility of a patient. To elucidate what essentially contributes to the onset and progression of IPAH, we investigated factors playing an important role in IPAH by searching discrepant or controversial expression patterns between our murine model and those previously published for human IPAH. We employed the mouse model, which induced muscularization of pulmonary artery leading to hypertension by repeated intratracheal injection of *Stachybotrys chartarum*, a member of nonpathogenic and ubiquitous fungus in our envelopment.

**Methods:**

Microarray assays with ontology and pathway analyses were performed with the lungs of mice. A comparison was made of the expression patterns of biological pathways between our model and those published for IPAH.

**Results:**

Some pathways in our model showed the same expression patterns in IPAH, which included bone morphogenetic protein (BMP) signaling with down-regulation of BMP receptor type 2, activin-like kinase type 1, and endoglin. On the other hand, both Wnt/planar cell polarity (PCP) signaling and its downstream Rho/ROCK signaling were found alone to be activated in IPAH and not in our model.

**Conclusions:**

Activation of Wnt/PCP signaling, in upstream positions of the pathway, found alone in lungs from end stage IPAH may play essential roles in the pathogenesis of the disease.

## Background

Pulmonary hypertension is a hemodynamic state characterized by elevation of the mean pulmonary arterial pressure leading to right ventricular (RV) failure and premature death. Pulmonary arterial hypertension (PAH) affects the small muscular arteries and arterioles in the lung and is histologically characterized by endothelial and smooth muscle cell proliferation, medial thickening, and thrombosis *in situ*. Idiopathic pulmonary arterial hypertension (IPAH), one of 6 subcategories proposed by Dana Point Classification [1], accounts for approximately half of PAH cases [2] and up to 40% of patients with no family history carries mutations in the bone morphogenetic protein receptor type 2 (BMPR2) gene [1]. 7% of patients with IPAH has a family history [3], and about 70% of these have long been recognized and are usually due to mutations in BMPR2, or much less commonly, 2 other members of the transforming growth factor superfamily, activin-like kinase type 1 (ALK1) and endoglin (ENG) [1]. While BMPR2 mutation strongly predisposes to IPAH, only 20% of mutation carriers develop a clinical disease [4]. This finding suggests that the development of IPAH first requires a genetic susceptibility, followed by one or several secondary triggering factors such as modifier genes and some sort of stimulus [5]. However, the pathogenesis of IPAH remains unclear.

To elucidate the pathophysiology of IPAH, we conducted genome-wide analysis of RNA expression profiles in lungs obtained from the murine model, which showed a favorable reproducibility to remodel pulmonary arteries induced by inoculation of *Stachybotrys chartarum*, an ubiquitous fungus in the surrounding environment [6]. This was followed in the study by exploration for factors playing a significant role in the onset of IPAH by searching discrepant or controversial expression patterns between in the model and those in IPAH previously published.

## Methods

### Detection of *S*. genes in the human lung tissue of IPAH patients and controls by nested polymerase chain reaction (PCR)

Upon getting the informed consent from patient’s family, a part of lung tissue obtained at autopsy from 9 children (under 18 years of age) with IPAH and 9 age-matched controls for detection of *S*. DNA in the tissue of human lung. It had been confirmed that none of them had inflammatory changes or other kind of infection in lungs by histopathological examination before DNA preparation. This protocol was approved by ethical committee of Toho University School of Medicine (# 20029). Using DNA isolated from the lung tissue, nested PCR and gel electrophoresis were performed in the usual manner. The following primers were used for identification of *S*. gene from autopsied lungs [7,8]: a primary primer of a two-step nested assay, forward primer S-Chart-tri5-F2-5^′^-TACACCAGGGAGGAGCGTGTT-3^′^ and reverse primer S-Chart-tri5-R2-5^′^-GCCGACAATGGTTCGAAGGGA-3^′^ produced a product of 138 bp. For the nested PCR primers, forward primer S-Chart-tri5-F2n-5^′^-GAAAATCTCCAGTATGCCT-3^′^ and reverse primer S-Chart-tri5-R2n-5^′^-AGCCTCCAGTCTCTTGGGG-3^′^ produced a product of 96 bp. For β-actin, the control for DNA quality, a two-step nested assay was also used with the following primers: forward primer B3-5^′^-ACACAACTGTGTTCACTAGC-3^′^ and reverse primer B4-5^′^-CAACTTCATCCACGTTCACC-3^′^ produced a product of 110 bp. For the nested PCR primers, forward primer b-globin-nestF34-52-5^′^-AACCTCAAACAGACACCAT-3^′^ and reverse primer b-globin-nestR103-85-5^′^-TTGCCCCACAGGGCAGTAA-3^′^ produced a product of 70 bp. The PCR amplification was performed as follows: initial denaturation at 94°C for 2 min, followed by 35 cycles of denaturation at 94°C for 30 s, annealing at 55°C for 30 s, with an extension at 72°C for 30 s, and a final extension at 72°C for 7 min. The second-round PCR reactions were performed in a manner identical to that applied for the first strand PCR, except for using different sets of primers. The PCR products were analyzed by electrophoresis on an agarose gel stained with ethidium bromide upon preparation.

### Fungal preparation and intratracheal injection

*S*. *chartarum* (IFM 53637), which produces trichothecene mycotoxins, was isolated from house dust in Japan, and has been stored in the culture collection of the Medical Mycology Research Center, Chiba University. The fungus was grown on potato dextrose agar (PDA) slants for 3 weeks at 25°C. Spores were collected in RPMI1640 medium (Sigma, St. Louis, MO, USA) and the concentration was adjusted to 4 × 10^5^ spores / ml. Spore concentrations and appearance of the suspension were evaluated under light microscopy before use. Six-week-old male ddY mice (Tokyo Laboratory Animals Science, Tokyo, Japan) were employed in this study. Mice were lightly anaesthetized with an intraperitoneal injection of ketamine (65 mg/kg BW) and xylazine (13 mg/kg BW). Their mean weight was 27.4 ± 1.21 g. The mice were placed in a supine position and a 24 G intravascular catheter (Insyte-W; Becton-Dickinson, Sandy, UT, USA) was then inserted intratracheally. The spore suspension (25 μl / mouse) containing 1 × 10^4^ spores was injected through the catheter into the trachea of each mouse 12 times at 4–5 day intervals for 8 weeks (n = 3) as described previously [6]. Control mice (n = 3) were injected with the same volume of RPMI-1640 medium rather than the spore suspension. All mice were cared for in accordance with the rules and regulations set out by the Prime Minister’s Office of Japan. Animal protocols were approved by the Special Committee on Animal Welfare of Chiba University. (DOU: 21–65).

### Histopathology and morphometric analysis of pulmonary arteries

Mice were sacrificed using by an overdose of diethyl ether inhalation 7 days after the last injection. Lungs were removed and fixed with a 10% formaldehyde solution, embedded in paraffin, cut into 3 μm-thick sections, and stained with hematoxylin and eosin for histopathological examination. Elastic fiber was stained with Elastica-van Gieson staining (Muto pure chemicals, Tokyo, Japan). Morphometric analyses were performed to determine the luminal stenosis of the pulmonary arteries. At least 200 pulmonary arteries per lung section from each mouse were chosen at random and examined. Cross-sections of arteries observed in the section were used to measure the distance between the external elastic lamina, internal elastic lamina, and intravascular lumen. All images were analyzed using IMAGE J 1.36b software (National Institutes of Health, Bethesda, MD, USA). The stricture rate was therefore calculated. Morphometry measurements were performed according to the techniques described in Dail and Hammar’s Pulmonary Pathology [9]. The thickness of media was calculated by subtracting the distance between the internal elastic lamina from that of the external lamina, and the thickness of the intima was calculated by subtracting the distance between the intravascular lumen from that of the internal elastic lamina. The distance between the external elastic lamina of the artery was defined as the diameter. Arteries were divided into three groups according to diameter: 50 < μm, 50–100 μm, and &100 μm. Data are given as mean ± SD. Statistical analyses were performed using Mann-Whitney's U test. Differences were considered significant at P < 0.05.

### RNA isolation and quality identification

RNA was isolated from the whole lung homogenates for both microarray analysis and Real-time (RT) Quantitative PCR with the RNeasy Lipid Tissue Mini Kit (Qiagen, Alameda, CA, USA) according to the manufacturer’s instructions and stored at −80°C. Total RNA quality was assessed and confirmed using the Agilent Bioanalyzer 2100 (Agilent Technologies, Palo Alto, CA, USA) for visualization of the 28S and 18S rRNA bands. RNA concentration and purity were also assessed and confirmed using the UV spectrophotometer NanoDrop™ND-1000 (NanoDrop Technologies, Wilmington, DE, USA), which calculates 260/280 ratios.

### RNA preparation for microarray analysis

cDNA preparation and microarray analysis were conducted at Bio Matrix Research (Chiba, Japan) using the Affymetrix system (Santa Clara, CA, USA). Isolated total RNA (100 μg) was converted into double-stranded cDNA using 3^′^IVT Express kit (Affymetrix, Santa Clara, CA, USA), which was purified using a GeneChip Sample Cleanup Module (Affymetrix, Santa Clara, CA, USA). *In vitro* transcription reactions were performed using a GeneChip IVT Labeling Kit, which includes T7 RNA polymerase and biotin-labeled ribonucleotides. Biotin-labeled cRNA was purified using a GeneChip Sample Cleanup Module. The concentration of cRNA was calculated from light absorbance at 260 nm using a UV spectrophotometer. cRNA (15 mg) was then fragmented at 94°C in the presence of a fragmentation buffer (Affymetrix, Santa Clara, CA, USA). The labeled cRNA was purified, fragmented, and spiked with *in vitro* transcription controls.

### Microarray analysis

Mouse Genome 430 2.0 microarrays (Affymetrix, Santa Clara, CA, USA) were hybridized with 12.5 μg of cRNA. The array was incubated for 16 hr at 45°C, and automatically washed and stained with the GeneChip Hybridization, Wash and Stain Kit (Affymetrix, Santa Clara, CA, USA) on an Affymetrix GeneChip Fluidics station. The arrays were analyzed using the GeneChip Scanner 3000. All preparations were run on quality-controlled chips and had 3^′^/5^′^ signal ratios of less than 3. The expression value of the transcript was computed using Affymetrix® GeneChip® Command Console® Software (AGCC) with the MAS5 algorithm [10], in which the probabilities of the values of each transcript were indicated as the “Flag” Present (p ≼0 to <0.04), Marginal (p ≼0.04 to <0.06), and Absent (p ≼ 0.06 to <0.5). Further analysis was performed with probes that had a present call in all analyzed samples. For analysis, the data were normalized using GeneSpring® GX 10.0 (Agilent Technologies, Palo Alto, CA, USA) data-mining software, per-chip normalization to the 50th percentile of the measurements for the array, and per-gene by normalizing to the median measurement for the gene across all the arrays in the data set. In addition, fold changes were calculated by this software for each gene between the experimental groups and controls. Statistically significant differences were investigated by means of unpaired t-tests. Gene expression differences with p < 0.05 and at least a ±1.3-fold change were considered statistically significant. We employed the Biological Networks Gene Ontology (GO) tool BINGO [11] (http://www.psb.ugent.be/cbd/papers/BiNGO) to find statistically over- or under-represented GO categories in biologic data as the tool for GO analysis of the stored genes. The analysis was done using the ‘hyper geometric test’, and all GO biological process terms that were significant with P < 0.05 (after correcting for multiple term testing using Benjamin and Hochberg false discovery rate corrections) were selected as over-represented and under-represented. Furthermore, the open access and curated pathway database REACTOME [12] (http://www.reactome.org) was used to determine which events (reactions and/or pathways) were statistically overrepresented in a set of genes.

### RT Quantitative PCR for evaluating the microarray results

cDNA preparation and RT-PCR were performed at Bio Matrix Research (Chiba, Japan). 2 μg of isolated RNA was used for cDNA synthesis (40 μl) using a Superscript III First Strand Synthesis System (Invitrogen, Carlsbad, CA, USA). To evaluate the concentration and purity of cDNA, 260/280 ratios were calculated using the UV spectrophotometer NanoDrop™ND-1000 (NanoDrop Technologies, Wilmington, DE, USA). PCR was performed in a 15 μl reaction mixture containing 1 μl of sample cDNA, 0.75 μl of TaqMan® Gene Expression Assays, 7.50 μl of TaqMan Universal PCR Master Mix, and 5.75 μl of RNase / DNase free water (Applied Biosystems, Foster City, CA, USA). 15 μl of PCR reaction mix was transferred into a 384-well reaction plate. The primer sequences are given in Table 1. Gene expression was measured on a 7900HT Fast RT-PCR system (Applied Biosystems, Foster City, CA, USA) with cycle conditions of 50°C/2 min, 95°C/10 min, 95°C/15 sec, and 60°C/1 min (steps 3–4 were repeated 40 times). Assay results were collected and analyzed using SDS 2.2 software (Applied Biosystems, Foster City, CA, USA). Each value is the mean of three biological replicates. Data are given as mean ± SD. Statistical analyses were performed by non-paired t-tests. Differences were considered significant at P < 0.05.

**Table 1:** Primers used in RT-PCR

**Gene Symbol**	Assay ID*	**RefSeq**^†^	**Context Sequence**
Acvrl1	Mm03053695_s1	NM_009612.2	TTTGTGGGAGCACTTGGCCTGTGAC
Bmpr2	Mm01254942_m1	NM_007561.3	AGTATACAGATAGGTGAGTCAACAC
Ccl8	Mm01297184_g1	NM_021443.2	CCATGGAAGCTGTGGTTTTCCAGAC
Ccl9	Mm00441260_m1	NM_011338.2	AGATCACACATGCAACAGAGACAAA
Ear11	Mm00519056_s1	NM_053113.2	CACAACTCCGGCCAGTCATTATTCC
Eng	Mm00468256_m1	NM_001146348.1	AAAAAACACGTGCAGACTCTCCAGT
Gapdh^‡^	Mm99999915_g1	NM_008084.2	TGAACGGATTTGGCCGTATTGGGCG
Igj	Mm00461780_m1	NM_152839.2	TCCGAATTGTTGTCCCTTTGAACAA
Mmp12	Mm00500554_m1	NM_008605.3	AAGTTTTCAAGGCACAAACCTCTTC
Mmp13	Mm00439495_g1	NM_008607.1	GAACCACGTGTGGAGTTATGATGAT
Mmp19	Mm00491300_m1	NM_021412.1	TGGTGCTGGGGCCTCGTGGGAAGAC
Nos3	Mm01134921_g1	NM_008713.4	CGGCGTGCTGCGGGATCAGCAACGC
Pdgfa	Mm01205760_m1	NM_008808.3	GGAGGAGGAGACAGATGTGAGGTGA
Vegfa	Mm01281449_m1	NM_001025250.3	ACGTACTTGCAGATGTGACAAGCCA

*Taqman® Gene Expression Assays.

^†^NCBI Gene Reference: NCBI transcript ID detected by the assay.

^‡^GAPDH: Glyceraldehyde-3-phosphate dehydrogenase was used as an internal control.

### Literature search concerning gene expression pattern in IPAH

We used PubMed (http://www.ncbi.nlm.nih.gov/pubmed/) to search for previous studies published since 2000 that analyzed biological molecules with altered expression using lung samples isolated from IPAH, compared with normal control or secondary PAH. From microarray studies, we referred to open access data stored in Gene Expression Omnibus: GEO (http://www.ncbi.nlm.nih.gov/geo/) and identified the genes that were differentially expressed following the methods described in each study. GO and Pathway analysis were performed on the genes in the same way as we done on our data.

### Comparing expression patterns of molecules between IPAH and experimental model

The biological molecules reported in various studies were collected and divided into groups according to the gene ontology biological process and pathways using the Gene Ontology Annotation Database GOA [13] (http://www.ebi.ac.uk/GOA/), REACTOME [12], and KEGG [14] (http://www.genome.jp/kegg/). The expression pattern of each group in IPAH was compared with our PAH models.

## Results

### Detection of *S*. genes in human lung tissue from patients with IPAH and controls by nested PCR

**Figure 1:**
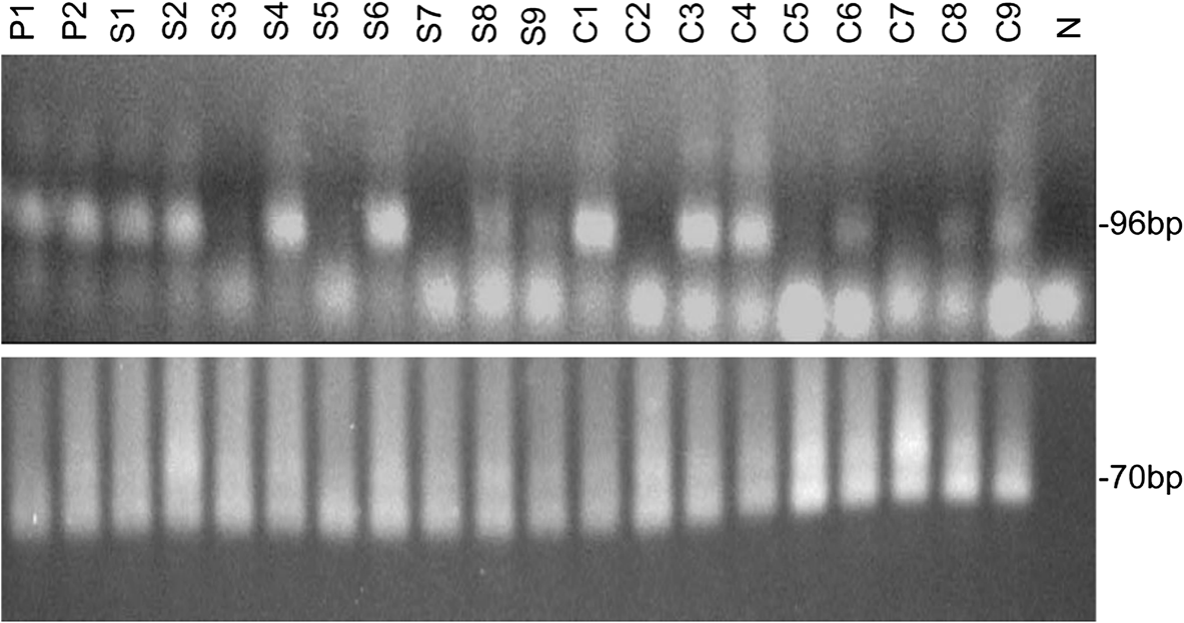
**Detection of *Stachybotrys chartarum *gene in the human lung tissue of IPAH patients and controls.** Agarose gel electrophoresis showed the amplification products after nested polymerase chain reaction for *S*. *chartarum* DNA using the standard assay. P1- P2: Positive control (96 bp and 70 bp); S1-S9: Children with IPAH; C1-C9: Age-matched control; N: Negative control (No DNA). *S*. *chartarum* and β-globin bands are 96 and 70 bp, respectively. 96 bp–specific band was detectable in P1-2, S1-2, S4, S6, S8-9, C1, C3-4, C6, C8-9.

*S*. *chartarum* DNA was detected in 6 of 9 lung samples among both two groups, children with IPAH and age-matched controls (Figure 1). There was no difference in the frequency (approximately 70%) of the detection in children, with or without of IPAH.

### Pathological findings in experimental PAH

**Figure 2:**
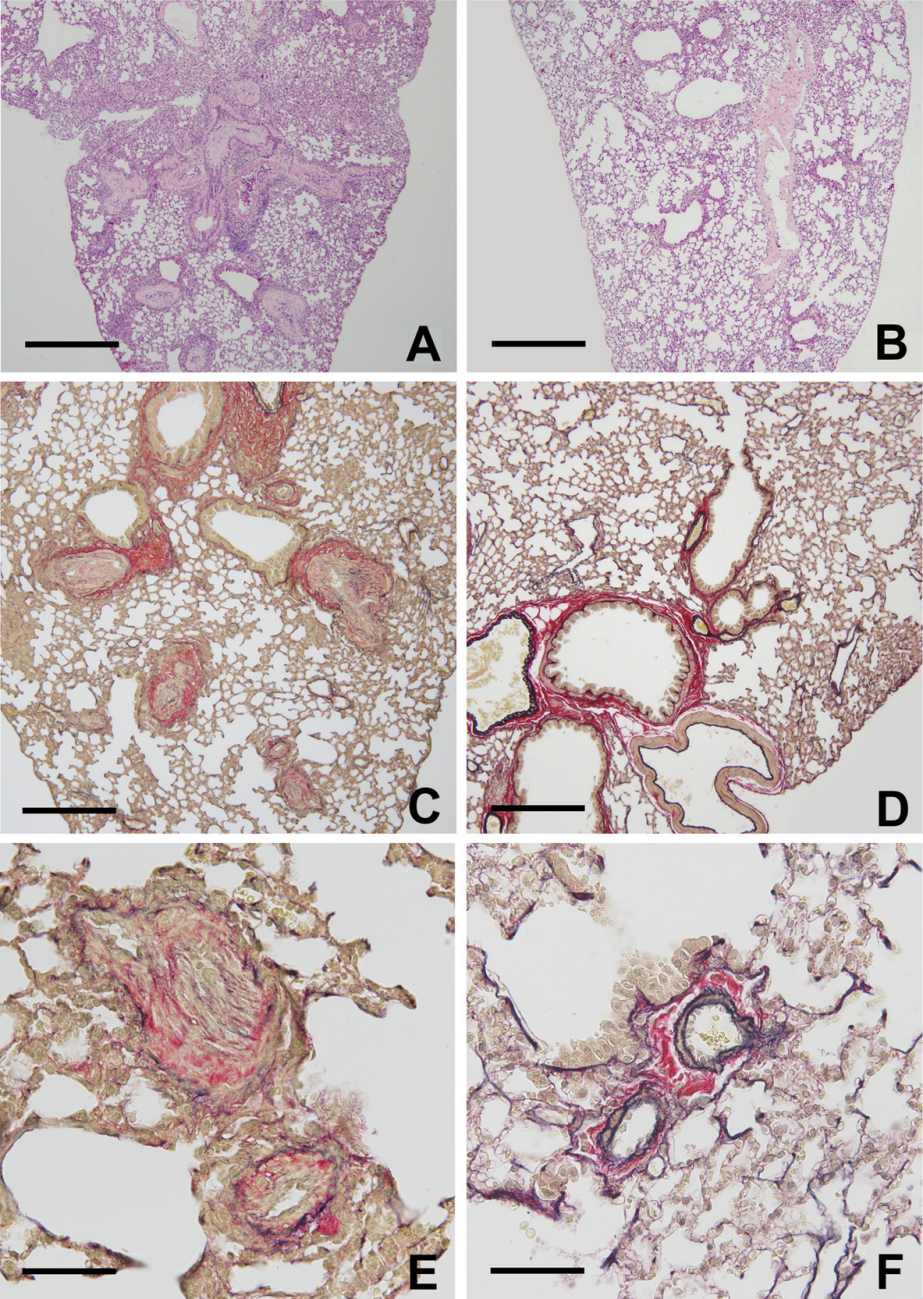
**Pulmonary vascular remodeling in *Stachybotrys chartarum*-exposed mice.** Peripheral pulmonary arteries in experimental group (**A**, **C**, and **E**) and control group (**B**, **D**, and **F**). **A** and **B**: hematoxylin and eosin double stain, **C**, **D**, **E**, and **F**: Elastic Van Gieson stain. (**A**) Pulmonary vascular thickening was present over the whole lung. (**C**) The arterial wall shows symmetrical thickening of the intima and media. (**E**) This vascular remodeling almost totally obliterates the lumens. (**B**, **D**, and **F**) Structure of the lung from control group is unaltered. Scale bars: 500 μm (**A**, **B**), 250 μm (**C**, **D**), and 50 μm (**E**, **F**).

**Figure 3:**
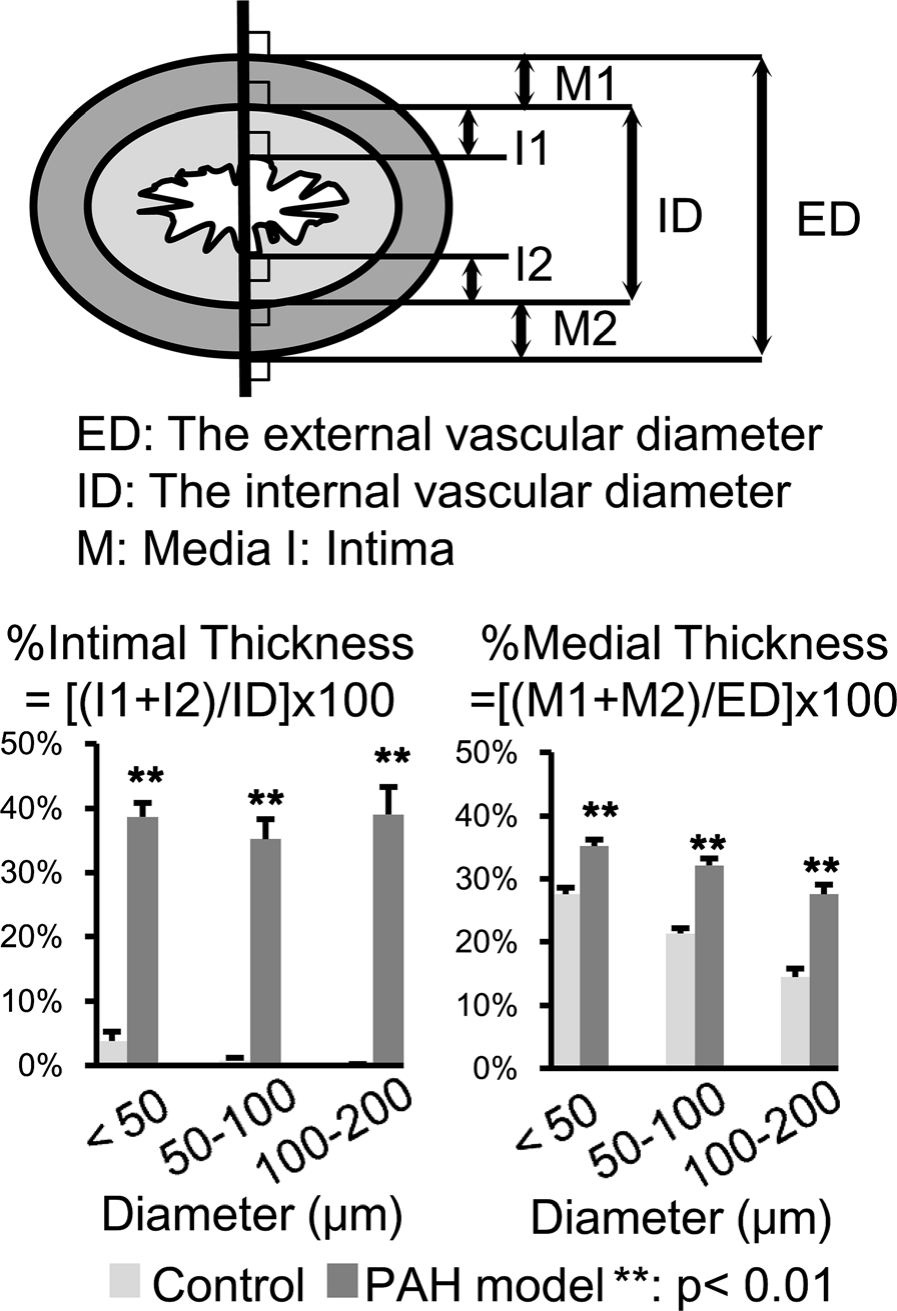
**Morphometric analysis evaluating luminal stenosis of pulmonary arteries.** Significant thickening of both intima and media were observed in the exposure group when compared with the control regardless of the size of vessels. Data are presented as means with standard error of the means. Statistical analyses were performed using Mann-Whitney's U test. A bar with ** is significantly different (P < 0.01).

Diffuse symmetric thickening of intima and media in the pulmonary artery was shown in the experimental group (Figure 2). The thickened intima and media were accompanied by proliferation of myointimal and smooth muscle cells, respectively. None of arteries showed alterations corresponding to necrosis, thrombosis, and plexiform lesions. The changes developed in arteries of small and medium-sized were mostly uniform. The thickened intima and media were statistically significant regardless of the size of vessels (Figure 3).

In this model, no venous canals were altered. None were found of components from injected fungus (spores and hyphae) and changes likely induced by the injection, such as perivascular cuffing and intraalveolar inflammatory exudates.

### Gene expression in the PAH model mouse

**Figure 4:**
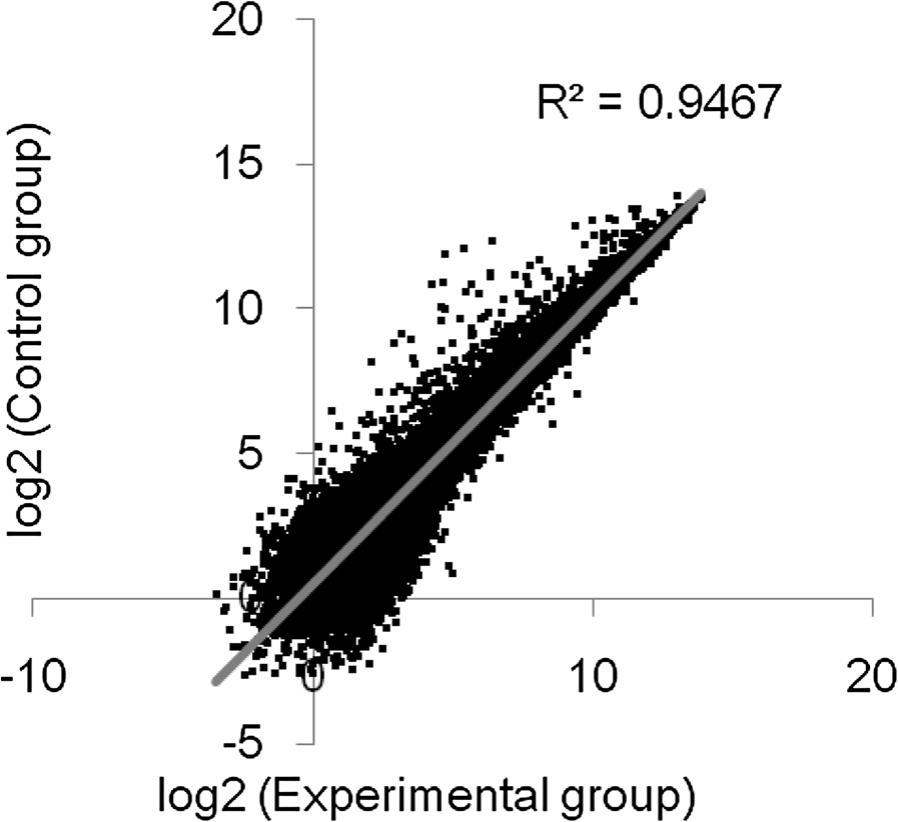
**Scatter plot of microarray dataset. Gene expression signal of control group (Y-axis) plotted against experimental group (X-axis).** The expression data are the average of three independent gene chips for each group.

Upon normalizing the expression values for the samples, the scatter plot of log intensity values was obtained as shown in Figure 4. 337 and 503 genes were found to exhibit up- and down-regulation from the lungs of mice with inoculation of the fungus in comparison to those from the control group. The most markedly up- and down- regulated genes are shown in Table 2. Down regulation of BMPR2, ALK1, and ENG was found in our PAH model. Down regulation of SMAD family member 6 expression was also observed, while the expressions of other genes involved in bone morphogenetic protein (BMP) signaling were unchanged. All the microarray data are MIAME compliant and the complete microarray data were deposited in GEO (accession number GSE23178). 696 biological process terms were detected by GO analysis in up-regulated genes. GO terms related to the immune system and cytokines accounted for about 80% of total terms. Among the remaining terms, estrogen receptor signaling pathway and serotonin transport/secretion were included. A statistically significant biological process was not found for down-regulated genes. Pathway analysis revealed that reactions and pathways related to the immune system, Janus kinase/signal transducers and activators of the transcription (JAK/STAT) pathway, and hemostasis etc. were detected (Table 3) in up-regulated genes. Additionally, vascular endothelial growth factor (VEGF), platelet-derived growth factor (PDGF), apoptosis, BMP signaling, etc. were detected in down-regulated genes.

**Table 2:** The top 10 most up-regulated and down-regulated genes

**Fold**-Change*	**Gene Symbol**	**Description**
[Up-regulated Genes]		
72.4	Ear11	Eosinophil-associated, ribonuclease A family, member 11
46.1	Mmp12	Matrix metallopeptidase 12
11.5	Rgs1	Regulator of G-protein signaling 1
11.4	Ccl9	Chemokine ligand 9
10.3	Ccl8	Chemokine ligand 8
10.0	Igj	Immunoglobulin joining chain
9.0	Igf1	Insulin-like growth factor 1
8.2	Gpnmb	Glycoprotein nmb
8.2	Ccl11	Chemokine ligand 11
8.2	Ccl22	Chemokine ligand 22
[Down-regulated Genes]		
4.2	Bex2	Brain expressed X-linked 2
3.1	Upk3b	Uroplakin 3B
2.9	Rrm2b	Ribonucleotide reductase M2 B
2.7	Lgals2	Lectin, galactoside-binding, soluble, 2
2.7	Acaa1b	Acetyl-Coenzyme A acyltransferase 1B
2.3	Shisa2	Shisa homolog 2
2.2	Ssr1	Signal sequence receptor, alpha
2.2	Kcnip4	Kv channel interacting protein 4
2.2	Msln	Mesothelin
2.1	Fam82b	Family with sequence similarity 82 member
2.1	Upk1b	Uroplakin 1B
2.1	Vldlr	Very low density lipoprotein receptor

*Fold change values are calculated by dividing the experimental group values by the control values.

**Table 3:** Events (reactions and/or pathways) associated with up-regulated and down-regulated genes

Event ID*	**Event Name**^**†**^	**P value**	**Input Genes in Event**^**‡**^	**Total No. of Genes**^**§**^
[Up-regulated Genes]				
98458	Immune System	1.5e-04	24	478
86456	Integrin alpha X beta 2 binds JAM-C	7.2e-03	2	6
108524	GPCR ligand binding	3.0e-02	13	322
104591	Eicosanoid ligand-binding receptors	3.8e-02	2	13
23802	Mouse JAK2 binds human common beta chain	5.1e-04	2	2
89750	Hemostasis	1.5e-03	19	400
33150	Endogenous sterols	3.4e-02	2	13
108993	Vitamin B2 metabolism	2.9e-03	2	4
111211	Stat5 tyrosine phosphorylation	4.9e-03	2	5
115537	IL7ra is phosphorylated on Y449	4.9e-03	2	5
81332	Ubiquitination of phospho-p27/p21	1.6e-02	2	9
107910	Synthesis, Secretion, and Deacylation of Ghrelin	2.0e-02	2	10
[Down-regulated Genes]				
92778	Axon guidance	3.9e-06	26	353
95727	Myogenesis	4.9e-02	2	13
90528	Signaling by VEGF	4.7e-04	4	14
100071	Integrin cell surface interactions	7.1e-03	9	123
82568	PDGF binds to extracellular matrix proteins	3.6e-02	4	45
118126	I-Smad competes with R-Smad1/5/8 for type I receptor	1.9e-02	2	8
91050	SOS phosphorylation and dissociation	7.3e-03	2	5
112933	Cell-Cell communication	1.7e-02	8	118
95483	Metabolism of nitric oxide	3.0e-02	2	10
84093	Ethanol oxidation	1.1e-03	3	8
79841	FMO oxidizes nucleophiles	2.3e-03	2	3
97703	Apoptotic execution phase	1.1e-02	5	49
107112	Aquaporin-mediated transport	8.0e-03	4	29
84730	MAPK targets/ Nuclear events mediated by MAP kinases	5.0e-02	3	30

*Reactome event accession number.

^†^Event name according to the Reactome Database.

^‡^Number of regulated genes in this model.

^§^Total number of genes involved in the event.

JAM: Junctional adhesion molecule, GPCR: G protein coupled receptors, JAK: Janus kinase, VEGF: Vascular endothelial growth factor, PDGF: platelet-derived growth factor, SOS: Son of Sevenless proteins, FMO: Flavin Monooxygenases, MAPK: mitogen-activated protein kinase, MAP: Mitogen-activated protein.

### Validation of microarray results by RT quantitative PCR analysis

RT-PCR was used to validate 14 selected genes that were induced or suppressed by the exposure. The correlation of fold changes in gene expression between the arrays and PCR is shown in Table 4. The results demonstrate completely the same gene expression pattern between both methods. The alterations of gene expressions were statistically significant in BMPR2, ENG, Vascular endothelial growth factor A, Platelet-derived growth factor alpha polypeptide, matrix metallopeptidase (MMP) 19, MMP12, eosinophil-associated ribonuclease A family member 11, and chemokine ligand 9.

**Table 4:** Quantitative PCR Validation of Microarray Expression data

**Gene symbol**	**Gene Name**	Fold Change (P value)*	
		**RT-PCR**	**Microarray**
Acvrl1	Activin A receptor, type II-like 1	0.7(0.07)	0.7(0.05)
Bmpr2	Bone morphogenic protein receptor, type II	0.6(0.04)	0.8(0.04)
Eng	Endoglin	0.5(0.05)	0.7(0.05)
Ccl8	Chemokine ligand 8	14.0(0.06)	10.3(0.04)
Ccl9	Chemokine ligand 9	9.5(0.03)	11.4(0.02)
Ear11	Eosinophil-associated, ribonuclease A family, member 11	76.0(0.04)	72.4(0.02)
Igj	Immunoglobulin joining chain	20.5(0.26)	10.0(0.05)
Mmp12	Matrix metallopeptidase 12	104.6(0.05)	46.1(0.02)
Mmp13	Matrix metallopeptidase 13	5.1(0.17)	5.7(0.05)
Mmp19	Matrix metallopeptidase 19	3.1(0.05)	2.9(0.05)
Nos3	Nitric oxide synthase 3, endothelial cell	0.7(0.09)	0.8(0.17)
Pdgfa	Platelet derived growth factor, alpha	0.7(0.01)	0.8(0.07)
Vegfa	Vascular endothelial growth factor A	0.6(<0.01)	0.7(0.04)
Gapdh^†^	Glyceraldehyde-3-phosphate dehydrogenase	1.0(<0.01)	0.9(0.46)

*Fold change values are calculated by dividing the experimental group values by the control values. Statistical analyses were performed by non-paired t-tests

^†^Gapdh was used as an internal control.

### Altered expression of biological molecules and genes in lungs from IPAH

The biological molecules reported in previous studies [15-28] were listed in Additional file 1. Microarray data of patients with IPAH and normal controls were referable from 3 individual studies [15,16,27]. The numbers of IPAH investigated in each study were 2 [16], 7 [27], and 18 [15], the mean age and its standard deviation were 44 ± 10 [16], 29 ± 16 [27], and 44 ± 18 [15], respectively. We analyzed the microarray data in each study and tried to extract the genes that showed common expression patterns through these three studies. However, there were few genes, and none of significant GO or pathways was identified among the previous reports regarding to IPAH.

### Comparing expression patterns of molecules between IPAH and experimental model

Events (reactions and/or pathways) and the expression patterns in IPAH extracted from the previous reports are listed with comparison to those resulted from our experimental model [15-28] (Table 5).

**Table 5:** Events (reactions and/or pathways) and the expression patterns in IPAH and in our PAH model

ID*	**Event Name**^**†**^	**IPAH**^**‡**^	**Present model**
map04630	JAK/STAT signaling	Up	UP
REACT_604	Hemostasis	Up	Up
GO:0030520	Estrogen receptor signaling pathway	Up	Up
GO:0007210	Serotonin receptor signaling pathway	Up	Up
GO:0001666	Response to hypoxia	Up	Not identified
map04310	Wnt/PCP pathway	Up	Not identified
REACT_19389	ROCK activation by Rho	Up	Not identified
REACT_16888	Signaling by PDGF	Up	Down
REACT_12529	Signaling by VEGF	Up	Down
GO:0042981	Regulation of apoptotic process	Up/Down	Down
REACT_12034	Signaling by BMP	Down	Down
REACT_6844	Signaling by TGF beta	Down	Not identified

*Accession number of Reactome, KEGG, and GO.

^†^Event name according to the Reactome, KEGG, and GO Database.

^‡^Expression pattern of biological molecules associated with the event in lung tissue obtained from patients with IPAH in previously published reports

JAK/STAT: Janus kinase-signal transducer and activator of transcription, PCP: planar cell polarity, ROCK: Ras Homolog- Associated Coiled-Coil-Forming Protein Kinase, Rho: Ras Homolog, PDGF: Platelet-derived growth factor, VEGF: Vascular endothelial growth factor, BMP: Bone morphogenetic protein, TGF: Transforming growth factor.

## Discussion

Since PAH is a progressive disease of unknown cause involving pulmonary arterial remodeling, characterized by relentless deterioration and death, intense investigations have been conducted in a variety of animal models [29] to know pathophysiology. The most commonly used were rats exposed to either hypoxia or monocrotaline, and newer models were introduced that involved modification of these approaches using rodents including transgenic mice [29,30].There were at least three genomewide studies conducting rat models among them, but little have been discussed with comparison to those in the human disease with pathway and GO analyses [31-33]. We have therefore aimed to elucidate a part of pathophysiology of PAH accompanied by pulmonary arterial remodeling with comparison in gene expression pattern between those previously known in end-stage IPAH and our murine model, of which muscularization in media and intima of pulmonary arteries was induced by inoculation of nonpathogenic fungus [6,34]. It was found that a large frequency of *S*. *chartarum* gene in the lung of both children with IPAH and age-matched controls in autopsy cases, whereas the prior histological examination had revealed no inflammatory changes with an association to fungal infection. The result suggests that the airway of human generally exposed by the ubiquitous fungus. Accordingly, unknown intrinsic factors may play a significant role in the onset of IPAH.

It was explored in the study to search the pathways which show common expression pattern among IPAH patients using microarray data previously published as well as up-loaded at the open access sites [15,16,27], but none were elucidated. A part of the result may reflect clinical differences in subjects such as age, gender, stage of the disease, and etc. On the other hand, the present murine model showed similar expression pattern of genes which has been generally accepted as some biological molecules associated with the pathogenesis of IPAH, such as BMP signaling [1,35,36]: BMPR2, ALK1, and ENG. Although some of previous genome-wide analyses [31-33] conducting rat models reported alterations of signaling pathway (Table 6), the present model revealed more favorable similarity in number of pathways expressing same manner to IPAH. However, the result from our study might be affected by recruited cellular components and/or reformed extracellular matrix, because RNA was extracted from the whole lung of the mice. Although RNA extraction was done one week after the latest inoculation of the fungus when no inflammatory change had been previously confirmed [6], in vitro work evaluating cells from the lungs of the mice would be required to look at functional alterations. In addition, whereas multiple time point’s evaluation could highlight the difference in pathophysiology of models of between our mice and rats previously published, we have evaluated once at the point when the most significant muscularization had been confirmed [6], since a priority in the study would have been set in comparing gene expression patterns between end-stage IPAH and our model. It has been generally accepted that the altered expression of BMP signaling was one of the important molecular reactions when pulmonary vascular remodeling developed as the response to some sort of stimulus [2,3]. This can be supported by the facts that only a few cases of BMPR2 mutation carriers developed clinical disease [4] and BMPR2-knockdown mice did not develop pulmonary artery medial hypertrophy, spontaneously [29]. Since remodeling of pulmonary artery in our model may be a sequel to inoculation of nonpathogenic fungus, down regulation of BMPR2 signaling should be simply understood as a consequence of muscularization which might be induced by alteration of signaling pathways at the upper stream. Besides BMP signaling, four more pathways known as identical expression patterns in IPAH were found in our PAH model. It has been suggested that inflammation might participate in the onset and propagation of pulmonary vascular remodeling in PAH via the JAK/STAT pathway [37,38], because elevated levels of inflammatory cytokines could trigger inflammation that is characteristic of PAH of both connective tissue disease-associated [39] and Virus-associated [40]. While PAH is up to threefold more prevalent in women than men [41], the increased expression of molecules associated with the estrogen signaling pathway are reported in both genders of patients with IPAH [15]. In addition, the evidence implicating serotonin has been discussed with correlation to the anorexigenic drugs aminorex and fenfluramine [42]. As with alteration of BMP signaling, the altered expression of these pathways would be simply associated with the consequence of pulmonary vascular remodeling developed as the response to some sort of stimulus. It might be better to understand as a sequel to vascular remodeling because that coagulation activity could be activated by various stimuli such as a cytokine and endothelial dysfunction [43].

**Table 6:** Summarized events (reactions and/or pathways) and the expression patterns in previously rat models

ID^*^	**Event Name**^**†**^	**Hypoxia-induced model**^**‡**^	**Hypoxia-induced model**^**§**^	**MCT-induced model**^**¶**^
map04630	JAK/STAT signaling	Not identified	Not identified	Not identified
REACT_604	Hemostasis	Down	Up/Down	Up/Down
GO:0030520	Estrogen receptor signaling pathway	Up	Not identified	Down
GO:0007210	Serotonin receptor signaling pathway	Up/Down	Not identified	Down
GO:0001666	Response to hypoxia	Up/Down	Up	Down
map04310	Wnt/PCP pathway	Not identified	Not identified	Not identified
REACT_19389	ROCK activation by Rho	Up	Not identified	Down
REACT_16888	Signaling by PDGF	Up	Up/Down	Down
REACT_12529	Signaling by VEGF	Not identified	Not identified	Down
GO:0042981	Regulation of apoptotic process	Up/Down	Not identified	Up/Down
REACT_12034	Signaling by BMP	Up/Down	Not identified	Not identified
REACT_6844	Signaling by TGF beta	Up	Up	Up

*Accession number of Reactome, KEGG, and GO.

^†^Event name according to the Reactome, KEGG, and GO Database.

^‡^Hypoxia-induced rat models reported by Moreno-Vinasco et al [31].

^§^Hypoxia-induced rat models reported by Kwapiszewska et al [32].

^**¶**^MCT-induced rat models reported by Vaszar et al [33].

JAK/STAT: Janus kinase-signal transducer and activator of transcription, PCP: planar cell polarity, ROCK: Ras Homolog- Associated Coiled-Coil-Forming Protein Kinase, Rho: Ras Homolog, PDGF: Platelet-derived growth factor, VEGF: Vascular endothelial growth factor, BMP: Bone morphogenetic protein, TGF: Transforming growth factor, MCT: Monocrotaline.

On the other hand, it emerged that some discrepant gene expression patterns between those previously known in IPAH and our model. Four pathways were identified as those altered alone in IPAH which comprised up-regulations of the Wnt/planar cell polarity (PCP) signaling pathway [27], the Ras homolog (Rho)/Rho- Associated Coiled-Coil-Forming Protein Kinase (ROCK) pathway [26,27], and the hypoxia response pathway [23,24], and down-regulations of the transforming growth factor beta (TGFB) signaling pathway [16]. Both PDGF signaling pathway [25] and VEGF signaling pathway [15,26] were found as those up-regulated in IPAH and down-regulated in this model. Interestingly, not detected was a pathway showing the reverse pattern.

Among these various pathways, we would focus on the Wnt/PCP pathway as the essential pathway of pathogenesis in IPAH because this pathway is presently known to be in the upper levels of hierarchical pathways regulating other related pathways [12-14]. Laumanns et al. reported that microarray analysis of lung tissue from patients with IPAH demonstrated the contribution of this pathway to the pathogenesis of IPAH [27]. It has been reported Wnt family of signaling proteins is essential for organ development in general, and lung morphogenesis in particular [44]. Especially, the PCP pathway signals through activation of the Rho/ROCK signaling pathway are implicated in cytoskeletal organization and epithelial cell polarity. In addition, this pathway has been shown to be required for normal lung development, and reports are beginning to emerge of links between PCP pathways and lung disease [45]. Besides IPAH, PCP gene expression changes were observed within isolated pulmonary vasculature in patients with pulmonary fibrosis. Studies of patients with IPAH have shown significant up-regulation of PCP signaling [27] and down-regulation of TGFB signaling [16]. Investigations have also shown inhibition of canonical Wnt signaling by PCP ligands [45], crosstalk between canonical Wnt signaling and TGFB signaling [46], and recruitment of both canonical and non-canonical Wnt pathways are required in BMP2 mediated angiogenesis in human pulmonary artery endothelial cells [47]. A part of results from the present study, no change of pathways around this system can support that altered interaction between the canonical Wnt pathway and the TGFB signaling pathway play an essential role for human disease and further clarification is expected of the role of the PCP pathway in the pathogenesis of IPAH. On the other hand, some previous reports conducted human disease reported the Rho/ROCK signaling pathway is also activated, but it is known that many other stimuli can activate this system [48]. An activation of this pathway may conduct to smooth muscle cell proliferation in pulmonary artery [49], we wish to understand this event as secondary episode to alteration in PCP signaling pathways.

Although, the details in the mechanism is unclear, various factors (e.g., Hypoxia, inflammation, and shear stress) are known as the inducer of growth factors such as VEGF and PDGF [50,51]. Accordingly, their activation may not be essential because they usually play at the lower level of hierarchical pathways, which are activated by many sorts of stimuli.

## Conclusion

Discrepancy in gene expression pattern between this model and the human disease previously reported suggests that activation of Rho/ROCK signaling via Wnt/PCP signaling plays an essential role in pathogenesis of IPAH.

## Competing interests

Dr. Shibuya reports receiving research grants from Pfizer Inc., Janssen Pharmaceutical K.K., Dainippon Sumitomo Pharma Co., Astellas Pharmaceutical Company, Taiho Pharmaceutical, and Pola pharma. All authors declare that they have no conflict of interest.

## Authors’ contributions

KS designed the study, participated in the data collections and the interpretation of the results, and drafted the manuscript as the first author. YO carried out the experiments and the histopathological evaluation and revised the manuscript as an equal contributor of the present study. EO, HK, and MS carried out the experiments and participated in the data collections. HN and TI carried out the histopathological evaluation and performed the statistical analysis. DS, NT, and TN carried out the histopathological evaluation and prepared figures. MW participated in the interpretation of the results, revised the manuscript, and gave final approval to the manuscript as a corresponding author. TS conceptualized the study and advised the first author on pulmonary hypertension as a clinical doctor. KK conceptualized the study and helped to draft the manuscript. KS conceptualized and designed the study, carried out histopathological and statistical evaluation, and revised the manuscript as a last author. All authors contributed to conceptualizing and writing this study. Furthermore, all authors read and approved the final manuscript.

## Supplementary Material

Additional file 1 Expression pattern of biological molecules in lung tissue obtained from patients with IPAHand related pathways. Click here for file
