# Supplementary material for: Gene expression analysis of a murine model with pulmonary vascular remodeling compared to end-stage IPAH lungs
Source: Respir Res. 2012 Nov 17;13(1):103. doi: 10.1186/1465-9921-13-103 (PMC3545891; doi:10.1186/1465-9921-13-103)
Supplement: Additional file 1 — Expression pattern of biological molecules in lung tissue obtained from patients with IPAHand related pathways. [file 1465-9921-13-103-S1.pdf]

Additional file 1. Expression pattern of biological molecules in lung tissue obtained from patients with IPAH and related pathways. Continued on next page.

| [Pathway Name, ID*] | Regulation | Authors |
|---|---|---|
| [ Signaling by BMP, REACT_12034] | | |
| BMPR2: bone morphogenetic protein receptor, type II | Down | Rajkumar R[15] |
| [ Signaling by TGF beta, REACT_6844] | | |
| TGFBR2: transforming growth factor, beta receptor 2 | Down | Fantozzi I[16] |
| TGFBR1: transforming growth factor, beta receptor 1 | Down | Fantozzi I[16] |
| [Jak-STAT signaling, map04630] | | |
| CCL2: chemokine ligand 2 | Up | Sanchez O[17] |
| IL15: interleukin 15 | Up | Masri FA[18] |
| Stat3: signal transducer and activator of transcription 3 | UP | Masri FA[18] |
| IL13RA2: interleukin 13 receptor, alpha 2 | Up | Hecker M[19] |
| MIR204: microRNA 204 | Down | Courboulin A[20] |
| [Hemostasis, REACT_604] | | |
| PF4: platelet factor 4 | Up | Rajkumar R[15] |
| F2RL3: coagulation factor II receptor-like 3 | Up | Rajkumar R[15] |
| P2RY1: purinergic receptor P2Y | Up | Rajkumar R[15] |
| ITPR1: inositol 1,4,5-trisphosphate receptor | Up | Fantozzi I[16] |
| TEK: endothelial-specific receptor tyrosine kinase | UP | Dewachter L[21] |
| TRPC6: transient receptor potential cation channel, subfamily C, member 6 | UP | Yu Y[22] |
| [Response to hypoxia, GO:0001666] | | |
| MMP2: matrix metallopeptidase 2 | UP | Lepetit H[23] |
| SLC6A4: solute carrier family 6, member 4 | UP | Eddahibi S[24] |
| [Signaling by PDGF, REACT_16888] | | |
| PDGFB: platelet-derived growth factor beta polypeptide | Up | Perros F[25] |
| PDGFRA: platelet-derived growth factor receptor, alpha polypeptide | UP | Perros F[25] |
| PDGFRB: platelet-derived growth factor receptor, beta polypeptide | Up | Perros F[25] |
| [Signaling by VEGF, REACT_12529] | | |
| VEGFA: vascular endothelial growth factor A | Up | Rajkumar R[15] |
| PKCA: PRKCA protein kinase C, alpha | Up | Do e Z[26] |
| PKCD: PRKCA protein kinase C, delta | Up | Do e Z[26] |

* Accession number of Reactome, KEGG, and GO.

Additional file 1. Expression pattern of biological molecules in lung tissue obtained from patients with IPAH and related pathways. Continued from previous page.

| [Pathway Name, ID*] | Regulation | Authors |
|---|---|---|
| [Wnt/PCP pathway, map04310] | | |
| WNT11: wingless-related MMTV integration site 11 | Up | Laumanns IP[27] |
| AKT1: v-akt murine thymoma viral oncogene homolog 1 | Up | Laumanns IP[27] |
| DAAM1: dishevelled associated activator of morphogenesis 1 | Up | Laumanns IP[27] |
| [ ROCK activation by Rho, REACT_19389] | | |
| ROCK1: Rho-associated, coiled-coil containing protein kinase 1 | Up | Do e Z[26] |
| ROCK2: Rho-associated, coiled-coil containing protein kinase 2 | Up | Do e Z[26] |
| RHOA: ras homolog gene family, member A | Up | Laumanns IP[27] |
| [Estrogen receptor signaling pathway, GO:0030520] | | |
| ESR1: estrogen receptor 1 | Up | Rajkumar R[15] |
| [ Serotonin receptor signaling pathway, GO:0007210] | | |
| HTR2B: 5-hydroxytryptamine receptor 2B | UP | Fantozzi I[16] |
| [Regulation of apoptotic process, GO:0042981] | | |
| Tmod3: tropomodulin 3 | Up | Rajkumar R[15] |
| E2f1: E2F transcription factor-1 | Up | Rajkumar R[15] |
| Mybl1: A-myb myeloblastosis viral oncogene homolog 1 | Up | Rajkumar R[15] |
| BcL2: B cell leukemia/lymphoma 2 | Up | Masri FA[18] |
| Mcl1: myeloid cell leukemia sequence 1 | Up | Masri FA[18] |
| TIMP1: tissue inhibitor of metalloproteinase 1 | Up | Lepetit H[23] |
| HOXA5: homeobox A5 | Up | Golpon HA[28] |
| Ncoa2: nuclear receptor coactivator 2 | Down | Rajkumar R[15] |
| Ppp2ca: protein phosphatase 2, catalytic subunit, α-isoform | Down | Rajkumar R[15] |
| PTGS2: prostaglandin-endoperoxide synthase 2 | Down | Rajkumar R[15] |
| PPP1R15A: protein phosphatase 1, regulatory subunit 15A | Down | Fantozzi I[16] |

* Accession number of Reactome, KEGG, and GO.
